# Health literacy, nutrition, complementary medicine and their associations with life satisfaction in cancer patients: a cross-sectional study

**DOI:** 10.1186/s12885-026-15848-z

**Published:** 2026-03-13

**Authors:** Luise Kiefer, G. Ciarlo, F. Ostendorf, E. Hintze, J. Hübner

**Affiliations:** 1 Department of Internal Medicine II, Hematology and Medical Oncology, University Hospital Jena, Friedrich Schiller University Jena, Am Klinikum 1, 07747 Jena, Germany; 2 Medical Clinic 1, University Hospital Frankfurt, Theodor-Stern-Kai 7, 60590 Frankfurt am Main, Germany

**Keywords:** Cancer patients, Life satisfaction, Health literacy, Nutrition-related symptoms, Weight loss, Complementary and alternative medicine (CAM), Patient-reported outcomes

## Abstract

**Background:**

Life satisfaction is a key patient-reported outcome in psychosocial oncology. Yet, how health literacy, nutritional behavior, eating-related symptoms, weight changes, and CAM use together influence life satisfaction remains poorly understood.

**Methods:**

We conducted a multicenter cross-sectional survey of 316 German oncology patients, assessing life satisfaction (L-1), health literacy, current food intake, eating-related symptoms, dietary frequency, weight change and CAM use. Analyses included descriptive statistics, Spearman correlations, nonparametric tests, and multivariate regression.

**Results:**

Subjective health status emerged as the strongest independent predictor of life satisfaction. Higher food intake and fewer eating-related symptoms were also beneficial. Moderate unintentional weight loss (below a high threshold) was associated with somewhat higher self-perceived life satisfaction, whereas more pronounced weight loss (> 10 kg) related to lower life satisfaction. Health literacy showed a modest correlation with life satisfaction in unadjusted analyses, but lost independent significance in multivariate models, suggesting mediation via other factors. Complementary and alternative medicine use was not associated with life satisfaction. The final predictive model explained about 35.1% of the variance in life satisfaction.

**Conclusion:**

These findings suggest that subjective health perceptions and nutritional factors are central drivers of life satisfaction in cancer patients, whereas the effect of health literacy appears to be mediated indirectly. The results support a holistic, patient-centered approach addressing subjective health, nutrition, and health literacy to improve psychosocial outcomes and life satisfaction.

**Supplementary Information:**

The online version contains supplementary material available at 10.1186/s12885-026-15848-z.

## Background

Life satisfaction in individuals with chronic illnesses is increasingly considered as a relevant outcome in patient-centered healthcare. In the context of cancer, diseases are often accompanied by substantial physical, psychological, and social distress. The subjective experience of quality of life (QoL) is gaining growing relevance in clinical and supportive care [1]. Beyond classical medical parameters, patient-reported outcomes such as life satisfaction, functional status, and personal life appraisal are becoming central to treatment planning and ongoing care [2].

International studies show that life satisfaction in cancer patients is shaped by a complex interplay of medical and psychosocial influences [3]. Frequently discussed are aspects such as nutritional symptoms, appetite changes, and involuntary weight loss, which may have both physiological and psychosocial implications in the context of malignancy [4]. At the same time, growing attention is being directed toward health-related resources, particularly individual health literacy – defined as the ability to access, understand, evaluate, and apply health-related information in everyday life [5]. While previous studies have often focused on individual domains of influence, the present investigation aims to jointly explore psychosocial, perceptual, and behavior-related characteristics in terms of their relevance to life satisfaction. Particular attention is given to health-related behavioral and cognitive domains, such as health literacy, nutritional habits, and the personal use of CAM factors, that have been addressed in earlier research but rarely examined together or in terms of their potential interactions with subjective life satisfaction. This cross-sectional study therefore seeks to identify which individual characteristics are associated with overall life satisfaction among people living with cancer. The results aim to help identify modifiable influencing factors and contribute to a more holistic approach to oncological care.

## Methods

### Study design

This study was designed as a prospective, multicenter, cross-sectional investigation. Data collection was conducted at the University Hospital Jena as well as at eleven additional oncology centers across Germany. The survey period extended from April 1, 2024, to November 30, 2024.

### Study participants

Questionnaires were distributed to patients at the University Hospital Jena and mailed to the other participating oncology centers. The participating institutions were asked to ensure that a sufficient number of questionnaires reached eligible patients. Inclusion criteria for the target sample included a current or past diagnosis of cancer, a minimum age of 18 years and sufficient proficiency in the German language to complete the questionnaire independently and anonymously. All questionnaires were paper-based and filled out manually by the patients. The study design aimed to ensure low-threshold access to participation for as many patients as possible. In total, 316 patients fulfilled the inclusion criteria and returned valid questionnaires.

The study size was therefore determined pragmatically by the number of participants enrolled during the recruitment period.

### Questionnaire

Data collection was based on a structured, paper-based questionnaire specifically developed for the present cross-sectional study. The aim was to provide a clear assessment of aspects of integrative oncology and CAM such as health literacy, spirituality, physical activity, and nutrition by using validated scales as well as study-specific items. This approach was intended to capture the unique life situations, needs, and health-related behaviors of oncology patients. The estimated completion time was approximately 15 to 20 min.

The questionnaire was divided into eight thematic core sections.

First section: Sociodemographic and clinical information was gathered, including age, gender, educational attainment, marital status, time of initial cancer diagnosis, and tumor entities. Treatment status was differentiated according to current and past therapeutic modalities (e.g., surgery, chemotherapy, radiotherapy). The respective items were presented as multiple-choice questions and supplemented with open-text fields for individualized responses.

Second section: Subjective health literacy was assessed using an eleven-item scale, developed in alignment with the internationally established HLS-EU-Q model [5]. The assessment covered four core dimensions:

- Active information seeking.
- Comprehension of health-related information.
- Critical appraisal of medical content.
- Application of such information in coping with the disease.

Items were rated on a four-point Likert scale ranging from “never” (1) to “always” (4). The operationalization was based on the conceptual framework of the European Health Literacy Survey, which defines health literacy as the ability to access, understand, appraise, and apply health-related information in everyday life [5].

Third section: The use of CAM was assessed. Eleven predefined CAM modalities were surveyed, including vitamin D supplementation, other vitamin or micronutrient supplements, herbal preparations, homeopathy, mistletoe therapy, yoga or Tai Chi, acupuncture, meditation or mindfulness-based practices, special diets, probiotics, and other CAM approaches specified by free-text responses. Items were presented as binary yes/no questions.

Fourth section: Physical activity and exercise were assessed, including retrospective and current movement behaviour. Participants were asked whether and which types of physical activity they engaged in before diagnosis and whether they currently remained physically active. Additional items captured awareness of cancer-specific exercise groups and the general desire to increase physical activity. Expectations regarding physical exercise were assessed using 18 items covering physical, psychological, and social goal dimensions. All items were rated on a seven-point Likert scale ranging from 1 (“does not apply at all”) to 7 (“fully applies”). A detailed overview of the item content is provided in Table 1. Furthermore, affective attitudes toward physical activity were measured using four seven-point semantic differential scales (e.g., “not relaxed” to “extremely relaxed”).

**Table 1:** Overview of the 18 items assessing expectations regarding physical activity

Domain	Item content (summary)
Physical health goals	Fitness improvement, health maintenance, symptom reduction
Psychological goals	Stress reduction, feeling balanced, immediate well-being
Weight and body-related	Weight regulation, body shape, body experience
Performance and competence	Physical challenge, performance comparison, capability
Experiential goals	Enjoyment, excitement, experiencing nature
Social goals	Social interaction, maintaining contacts, meeting new people

Fifth section: Spiritual attitudes and sense of meaning were assessed using the validated FACIT-Sp-12 scale [6]. This 12-item scale captures three dimensions:

- Meaning.
- Peace.
- Faith.

Each subscale comprised four items, rated on a five-point Likert scale (0 = not at all to 4 = very much), referring to experiences during the past seven days.

Sixth section: General life satisfaction was measured using the L-1 scale developed by GESIS [7]. This unidimensional measure consists of a single-item question: “How satisfied are you with your life overall at the moment?”, rated on an eleven-point scale (0 = not at all satisfied to 10 = completely satisfied).

Seventh section: Nutrition was comprehensively assessed. Patients were asked to indicate their current dietary pattern (vegetarian, vegan, sugar-free, gluten-free, ketogenic, or mixed diet). Multiple responses were permitted, with a free-text option for individualized descriptions. General health status was rated on a five-point scale (1 = very good to 5 = very poor). Information on unintentional weight changes since cancer diagnosis was collected, including free entry of kilograms lost.

Current eating behaviour was assessed by:

- Food intake in recent weeks (four-point scale, 1 = more than usual to 4 = about half as much as usual).
- Portion size at the main meal, measured with a visual response scale of five symbolic plates (almost all – almost nothing).

Patients also reported eating-related symptoms, choosing from twelve multiple-choice options (e.g., dry mouth, nausea, bloating, diarrhoea, early satiety), supplemented with free-text fields.

Finally, dietary frequency was assessed for twelve food groups (vegetables, fruits, meat, fish, dairy products, grains, processed foods, nuts, etc.) over the past four weeks. Frequency was rated on a nine-point scale (1 = never/almost never to 9 = more than six times daily). This assessment was based on the principles of semi-quantitative Food Frequency Questionnaires (FFQs) [8].

### Statistical analysis

All statistical analyses were performed using IBM SPSS Statistics (Version 31). All tests were conducted two-sided with a significance level of α = 0.05. The choice of statistical procedures was based on the scale level of the respective variables as well as the results of normality testing (Shapiro–Wilk test, Kolmogorov–Smirnov test) and homogeneity of variances (Levene’s test). Descriptive statistics included means, standard deviations, and absolute as well as relative frequencies. For all descriptive analyses, percentages were calculated using valid cases only. To analyze bivariate associations, Spearman’s rank correlation coefficient (ρ) was applied. This non-parametric method chosen due to the absence of normal distribution and the partly ordinal level of measurement. To test group differences between two independent samples, the non-parametric Mann–Whitney U test was used in most cases, particularly for the variable’s life satisfaction and subjective health status, as these did not meet the assumption of normality. For comparisons involving more than two groups, the Kruskal–Wallis test was applied. When assumptions were met, one-way analyses of variance (ANOVA) were conducted. For inhomogeneous variances, Welch’s ANOVA with Games–Howell post hoc tests were used. Effect sizes were reported as Pearson’s r, Cohen’s d, or Eta² and classified according to Cohen’s thresholds. Chi-square (χ²) tests were additionally employed to assess associations between nominally scaled variables, such as gender-specific differences in tumor types and treatments. In cases of low cell counts (*n* < 5), interpretation was omitted, or the test was not reported. For significant chi-square results, Cramer’s V was provided as a measure of effect size. To identify relevant predictors of life satisfaction, multiple linear regression analyses were conducted. Predictor selection was based on theoretical assumptions and preliminary bivariate analyses. Various models included variables such as subjective health status, health literacy, categorized weight loss, current food intake, number of nutrition-related symptoms, use of CAM and time since diagnosis. In addition to regression coefficients (β) and p-values, explained variance (R², adj. R²) and multicollinearity indicators (VIF) were reported. Analyses were performed separately for the dependent variable general life satisfaction and additionally for subjective health status. All analyses followed a structured, theory-driven approach and were comprehensively documented with regard to statistical significance, effect sizes, and model quality.

## Results

### Sociodemographic and disease-related characteristics

The study included 316 oncology patients with a mean age of 61.8 years (SD = 11.8). Two thirds of the participants were female (65.2%) and one third were male (34.8%). Among the 300 patients who provided information on educational attainment, nearly three quarters (74.0%) reported secondary or higher education. The most frequently reported diagnoses were breast cancer (34.9%), colorectal cancer (14.4%), and lung cancer (10.9%). The mean duration of illness was 36.4 months (SD = 47.1). At the time of data collection, about half of the participants (51.1%) were receiving chemotherapy, whereas 22.4% had undergone surgery, 16.4% radiation therapy and 10.0% hormone therapy. Marked gender-specific patterns emerged: men were more likely to be receiving chemotherapy (68.8% vs. 41.6%), whereas women reported higher rates of hormone therapy (14.4% vs. 1.8%) and radiation therapy (20.3% vs. 9.2%). A comprehensive overview of all sociodemographic and clinical characteristics is presented in Table 2.

**Table 2:** Sociodemographic and clinical characteristics of the study sample (*N* = 316)

Characteristic	Total (*N* = 316)	Men (*n* = 110)	Women (*n* = 206)
Age (years), n, mean ± SD	289, 61.8 ± 11.8	100, 64.9 ± 11.9	189, 60.2 ± 11.4
Education ≥ secondary, n (%)	234 (76.0)	68 (62.9)	155 (77.5)
Marital status, n (%)			
Married	207 (67.0)	79 (73.1)	127 (63.2)
Other (single/divorced/widowed)	102 (33.0)	29 (26.9)	73 (36.3)
Time since diagnosis (months), n, mean ± SD	301, 36.4 ± 47.1	104, 40.2 ± 45.5	197, 34.4 ± 47.9
Cancer type, n (%)			
Breast	109 (34.9)	1 (0.9)	108 (53.2)
Colorectal	45 (14.4)	31 (28.4)	14 (6.9)
Lung	34 (10.9)	18 (16.5)	16 (7.9)
Prostate	13 (4.2)	13 (11.9)	0 (0.0)
Digestive tract (excl. colorectal)	39 (12.5)	27 (24.8)	12 (5.9)
Gynecologic	25 (8.0)	0 (0.0)	25 (12.3)
Other	53 (17.0)	21 (19.3)	32 (15.8)
Current treatment, n (%)			
Chemotherapy	159 (51.1)	75 (68.8)	84 (41.6)
Surgery	70 (22.4)	17 (15.6)	53 (26.1)
Radiation therapy	51 (16.4)	10 (9.2)	41 (20.3)
Hormone therapy	31 (10.0)	2 (1.8)	29 (14.4)

Percentages are calculated based on the number of participants with valid responses for each variable; denominators therefore vary across characteristics and sex

### Subjective health status

In the overall sample (*N* = 311), 2.3% of respondents rated their health status as “very good,” 39.2% as “good,” and 49.2% as “average.” A total of 8.0% assessed their health as “poor,” while 1.3% selected the category “very poor.” Gender-specific differences were not statistically significant, with men (9.3%) and women (9.0%) reporting similarly high proportions of “poor” or “very poor” subjective health. Subjective health status showed significant associations with various psychosocial and nutrition-related factors. Spearman’s correlation indicated a negligible-to-weak association between health literacy and subjective health status (rₛ = 0.186; *p* = 0.001). ANOVA also revealed significant differences in mean scores across subjective health groups (F (4,305) = 3.625; *p* = 0.007), which were confirmed by post hoc tests (Tukey-HSD, *p* = 0.007). A negligible-to-weak association was observed between subjective health status and the total number of reported nutrition-related symptoms (rₛ = 0.196; *p* = 0.001). Individual complaints such as fatigue (*p* < 0.001), early satiety (*p* = 0.044), nausea (*p* = 0.012) and diarrhea (*p* = 0.001) occurred significantly more often among those with poorer subjective health status. Various dietary habits were also related to health status. Nut consumption showed a negligible inverse association with subjective health status (rₛ = −0.126; *p* = 0.029).

Participants who reported following a sugar-free diet showed significantly lower health status values (*p* = 0.013), reflecting a more favorable self-assessment. In contrast, reduced recent food intake was linked to a negligible association with subjective health status (rₛ = 0.116; *p* = 0.043), indicating poorer perceived health. Moreover, significant differences were observed among respondents who reported a history of medication-based treatment (*p* = 0.039) or other previous therapeutic interventions (*p* = 0.019). These groups reported significantly worse health status than those without such histories. At the end of the bivariate analysis section, Spearman’s correlation indicated a weak inverse association between subjective health status and life satisfaction (rₛ = −0.351; *p* < 0.001). Thus, a lower self-assessed health status was associated with reduced life satisfaction. Within the multivariable linear regression model predicting life satisfaction (Table 3), subjective health status emerged as the strongest independent predictor (β = −0.456, *p* < 0.001). In addition, higher current food intake (β = 0.181, *p* = 0.031) and a greater burden of nutrition-related symptoms (β = −0.169, *p* = 0.048) remained independently associated with life satisfaction, while the full set of covariates entered, including time since diagnosis, did not materially alter these associations (Table 4). Overall, the primary model explained 35.1% of the variance in life satisfaction (R² = 0.351; adjusted R² = 0.302; F (8,106) = 7.173, *p* < 0.001; Table 3). A sensitivity analysis excluding subjective health status (Table 3) yielded a reduced model in which nutrition-related symptoms (β = −0.279, *p* = 0.003) and greater weight loss severity (β = 0.214, *p* = 0.019) remained independently associated with life satisfaction; the full set of included predictors, including time since diagnosis, is reported in Table 4. This sensitivity model explained 14.6% of the variance in life satisfaction (R² = 0.146; adjusted R² = 0.099; F (6,109) = 3.099, *p* = 0.008; Table 3).

**Table 3:** Multivariable linear regression models for life satisfaction: primary model including subjective health status and sensitivity model excluding subjective health status

Model	*N*	F(df)	*p*	*R*²	adj. *R*²
Primary model	115	7.173 (8,106)	< 0.001	0.351	0.302
Sensitivity model	116	3.099 (6,109)	0.008	0.146	0.099

N reflects complete cases for all variables included in each model (listwise deletion). The primary model included subjective health status as a predictor, resulting in a reduced sample size, whereas the sensitivity model excluded this variable

**Table 4:** Standardized regression coefficients for life satisfaction in primary and sensitivity models

Predictor	Primary: β (*p*)	Sensitivity: β (*p*)
Subjective health status	−0.456 (< 0.001)	—
Eating-related symptoms	−0.169 (0.048)	−0.279 (0.003)
Current food intake	+ 0.181 (0.031)	+ 0.163 (0.084)
Health literacy	−0.133 (0.101)	−0.158 (0.083)
Weight loss (grouped categories)	+ 0.151 (0.063)	+ 0.214 (0.019)
Use of CAM (overall use)	+ 0.102 (0.335)	+ 0.000 (0.996)
Time since diagnosis (months)	−0.082 (0.314)	−0.058 (0.526)

Values are standardized regression coefficients (β) with two-sided P values. Higher scores indicate worse subjective health status (1 = very good, 5 = very poor). Weight loss severity was analysed in three groups (1–5 kg, 6–10 kg, > 10 kg). N corresponds to complete cases for all variables included in each model (listwise deletion; see Table 3). Dashes indicate predictors not included in the respective model

### Life satisfaction as the primary outcome variable

General life satisfaction was fully assessed in 305 patients. The mean score was M = 7.03 (SD = 2.02), with a median of 8.00. The observed range extended from 1 to 10. A categorical analysis revealed that 11.1% of respondents reported low life satisfaction (1–4 points), 38.4% fell into the medium range (5–7 points), and 50.5% reported high life satisfaction (8–10 points). Tests for normality indicated significant deviations in both the Kolmogorov–Smirnov test (D = 0.195; *p* < 0.001) and the Shapiro–Wilk test (W = 0.901; *p* < 0.001). Based on these findings, nonparametric procedures were used for the bivariate analyses. In the gender-specific analysis, women (*n* = 199) showed a mean score of M = 6.93 (SD = 1.93), while men (*n* = 102) had a mean score of M = 7.26 (SD = 2.17). Life satisfaction showed a negligible inverse association with disease duration (months) (Spearman’s rₛ = −0.127, *p* = 0.030). In the following sections of the results, life satisfaction is presented as the dependent outcome variable in relation to thematically grouped predictor variables.

### The impact of health literacy on subjective life satisfaction

Complete data on health literacy were available for 315 patients. The overall mean score was M = 1.90 (SD = 0.44), with a range from 1.00 to 3.18. The gender-specific analysis showed no significant difference: women had a mean health literacy score of M = 1.91 (SD = 0.44; *N* = 202), while men had a mean of M = 1.88 (SD = 0.46; *N* = 109). Spearman’s correlation indicated a negligible inverse association between educational level and health literacy (rₛ = −0.137; *p* = 0.018; *N* = 311), indicating that higher levels of education were associated with lower health literacy scores, reflecting better health literacy. In addition, significant associations emerged with subjective health status: tumor patients with more favorable self-assessments showed better health literacy scores on average. The coefficient suggested a negligible positive association between subjective health status and health literacy (rₛ = 0.186; *p* = 0.001; *N* = 311). One-way analysis of variance also revealed significant differences in health literacy depending on health status (F (4,305) = 3.625; *p* = 0.007). Post hoc comparisons (Tukey HSD) confirmed lower health literacy scores in groups with more favorable subjective health. A significant association was also observed between health literacy and overall life satisfaction. Health literacy showed a negligible inverse association with life satisfaction (Spearman’s ρ = −0.187; *p* = 0.001; *N* = 303), with lower health literacy scores being associated with lower levels of life satisfaction. In the regression model predicting life satisfaction, the health literacy mean score was included as an independent predictor but did not show a significant effect within the full model (β = − 0.133; *p* = 0.101; Tolerance = 0.949; VIF = 1.053; *N* = 115).

### Nutrition as a determinant of life satisfaction

The assessment of current food intake (*N* = 302) revealed that the majority of cancer patients reported having eaten (almost) all of their usual portion at the time of the survey (61.9%). Approximately one-quarter consumed around half of a normal portion (24.5%), while smaller proportions reported having eaten only a quarter (8.9%) or nothing at all (4.6%). There was no significant gender difference in reported food intake (*p* = 0.232). No bivariate association with overall life satisfaction could be established. Spearman’s coefficient suggested no meaningful association between current food intake and life satisfaction (rₛ = −0.025; *p* = 0.670).

**Fig. 1:**
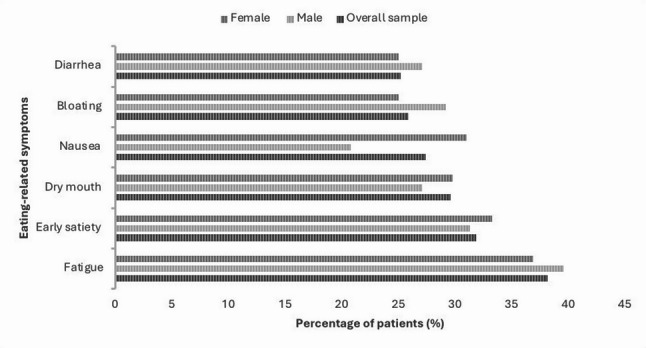
Percentage of male and female cancer patients reporting eating-related symptoms. For clarity, only the most frequently reported symptoms (> 10% of the sample) are displayed

In contrast, the multiple regression model, which simultaneously accounted for various influencing factors, showed a significant predictive effect of current food intake: higher levels of reported intake were associated with higher satisfaction scores (β = 0.181; *p* = 0.031). The multivariate analysis was based on *N* = 115 valid cases (Tolerance = 0.893; VIF = 1.120). In addition, symptom burden during eating was assessed, reflected by the number of reported nutrition-related symptoms. The frequency analysis showed that 315 individuals reported at least one symptom, with a mean of 3.44 symptoms per patient. Figure 1 illustrates the distribution of the six most frequently reported eating-related symptoms (≥ 10% of the sample), with fatigue, early satiety, and dry mouth being most common.

In the bivariate analysis, no significant association between the total number of symptoms and overall life satisfaction was found (rₛ = − 0.052; *p* = 0.369). Nevertheless, multiple regression revealed a significant effect: the total number of reported symptoms emerged as an independent predictor of life satisfaction (β = − 0.169; *p* = 0.048; Tolerance = 0.857; VIF = 1.166; *N* = 115). This association remained stable after controlling for other relevant factors. With regard to self-reported weight loss (*N* = 126), 40.6% of respondents indicated they had lost weight since their diagnosis. A more detailed analysis showed that greater weight loss was associated with lower levels of life satisfaction. In the bivariate analysis, weight loss (metric) showed a weak positive association with life satisfaction (Spearman’s rₛ = 0.212; *p* = 0.020). Additionally, significant group differences in life satisfaction were observed depending on the extent of weight loss (Kruskal-Wallis H = 7.616; *p* = 0.022), with particularly low satisfaction scores reported among patients who had lost more than 10 kg.

### Exploring the relationship between CAM use and life satisfaction

**Fig. 2:**
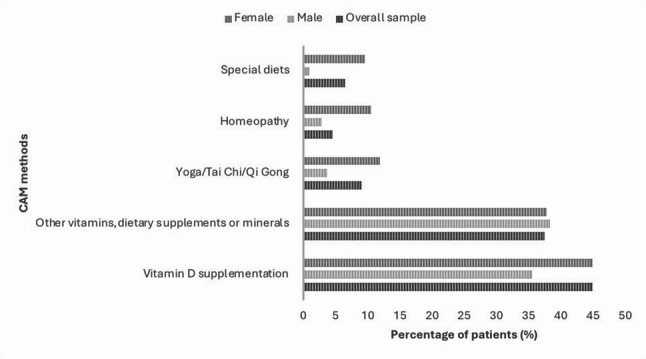
Percentage of male and female cancer patients using selected CAM methods (only methods reported by > 5% of the total sample are displayed)

A total of 191 out of 312 surveyed patients (61.2%) reported using at least one CAM method. Figure 2 shows the distribution of the most frequently used CAM modalities by sex, only CAM methods reported by more than > 5% of participants are displayed. The most commonly used approaches were vitamin D supplementation (*n* = 140; 44.9%) and the intake of other vitamins and dietary supplements (*n* = 117; 37.5%). Women were significantly more likely than men to take vitamin D supplements (χ² (1) = 5.723; *p* = 0.017). No significant differences in overall life satisfaction were found between users and non-users of individual CAM methods. The corresponding Mann-Whitney U tests yielded non-significant results for all methods examined, including vitamin D, other vitamins, herbal preparations, yoga/Tai Chi, homeopathy, and others (all *p* > 0.05). Similarly, among high-frequency users (≥ 4 CAM methods), no significant association with life satisfaction was observed (Spearman rₛ = − 0.131; *p* = 0.212; *n* = 93). In the multiple regression model accounting for various influencing factors on life satisfaction, the use of CAM methods likewise was not identified as a significant predictor (β = 0.102; *p* = 0.335).

## Discussion

The present cross-sectional study investigated factors associated with overall life satisfaction among individuals with an oncological diagnosis, focusing on health literacy, nutrition, and the use of CAM. Life satisfaction was chosen as the central outcome, as it represents a core patient-reported indicator in psychosocial oncology research [2]. The regression analysis yielded the strongest results when subjective health status was included as a predictor: subjective health status showed a consistent and robust association with life satisfaction. In addition, higher current food intake and lower symptom burden during meals were significantly related to better life satisfaction, suggesting that both physical and subjectively experienced factors are involved [2]. When subjective health status was excluded from the model, nutrition-related symptoms remained negatively associated with life satisfaction, and unintentional weight loss emerged as an additional factor. In this model, current food intake no longer reached statistical significance, which points to an indirect pathway mediated by perceived health [4]. Subjective health status had initially been considered a second outcome variable, but the analyses revealed that it was itself strongly determined by life satisfaction and could not be explained independently in the absence of psychosocial predictors [3]. For this reason, the discussion focuses exclusively on predictors that contributed to explaining life satisfaction and contextualizes these results within current literature, while also reflecting on implications for psychosocial and supportive care in cancer patients.

The findings underscore that subjective health status is the strongest determinant of life satisfaction in cancer patients. This observation has clinical as well as methodological implications, as it emphasizes the relevance of personal appraisal processes and coping strategies in chronic disease management. Cancer is not only a somatic condition but an existentially distressing experience, often associated with anxiety, loss of control, and an altered self-image, which directly affect psychological functioning and psychosocial outcomes [9]. In contrast to self-limiting acute illnesses, where subjective health perceptions play only a minor role, they are central to psychosocial QoL in long-term oncological trajectories. Ellis et al. [9], for example, demonstrated that it was not objective clinical indicators but subjective complaints and fatigue that primarily explained QoL in breast cancer patients. The present results thus confirm the pivotal role of subjective health status in shaping life satisfaction, supporting the notion that psychosocial outcomes and life satisfaction are more strongly tied to perceived health than to measurable clinical variables.

Beyond general health perceptions, eating-related symptoms emerged as significant contributors to life satisfaction. Meals are of central physiological and social importanc and symptoms such as appetite loss, early satiety, diarrhea, or fatigue can severely restrict not only nutritional intake but also enjoyment, participation in social life, and perceived vitality. The current findings therefore highlight the need to systematically address eating-related discomfort as part of supportive cancer care. These results are consistent with previous studies demonstrating that loss of appetite and fatigue are among the most prominent determinants of health-related QoL in cancer patients [9]. Addressing these symptoms through targeted interventions may help improve psychosocial outcomes, as they represent both physical burdens and psychosocial stressors that influence life satisfaction through multiple pathways.

Nutritional behavior itself also showed relevant associations. Greater current food intake was initially positively associated with life satisfaction, but this effect was largely explained by subjective health status. This indicates that eating behavior may influence life satisfaction indirectly by affecting patients’ perceptions of their health [10]. Reduced food intake could therefore lead to negative self-assessments of vitality and resilience, which in turn lower life satisfaction. When subjective health status was removed from the model, weight loss emerged as an independent predictor of life satisfaction, revealing a more nuanced picture. Interestingly, moderate weight loss (1–10 kg) was positively associated with life satisfaction, which may at first seem counterintuitive. One possible explanation is that, for certain subgroups such as overweight patients or those following specific dietary approaches (e.g., sugar-free or ketogenic diets), moderate weight loss may be interpreted as an indicator of successful self-regulation, improved self-efficacy, and healthier lifestyle behaviours. In line with this, Phillips et al. [11] showed that moderate post-diagnosis weight loss in breast cancer survivors was associated with reduced fatigue, anxiety, and stress, as well as improvements in physical self-worth and several domains of health-related quality of life. By contrast, pronounced and unintentional weight loss of more than 10 kg likely reflects disease progression, treatment side effects, or malnutrition, and is associated with declines in functional capacity, muscle mass, and overall resilience [12]. These patients may feel physically weaker, less autonomous, and less able to cope with their disease, which in turn reduces life satisfaction. Taken together, these findings point to the importance of weight stability and highlight that both the magnitude and the meaning of weight changes need to be considered in patient-centered care.

Neither the use of CAM nor individual health literacy showed a significant direct association with life satisfaction. More than 60% of participants reported CAM use, with vitamin supplements being most common, but no consistent benefits for life satisfaction were observed. This is consistent with prior research suggesting that CAM often serves as a supportive resource for coping and emotional stabilization rather than as a direct determinant of life satisfaction [13]. Similarly, no independent effect of health literacy on life satisfaction was identified in adjusted models. Nevertheless, participants with higher health literacy tended to assess their general health more favourably, suggesting that health literacy influences cognitive appraisal of health rather than global life satisfaction. This interpretation is consistent with prior studies linking higher health literacy with better health-related quality of life, mediated through reduced emotional distress, improved coping, and enhanced patient–provider communication [14–17]. In our study, the negative correlation initially observed between health literacy and life satisfaction did not persist in regression analysis after adjusting for subjective health status and other variables. This indicates that the influence of health literacy is largely indirect, shaping how patients interpret symptoms and manage health information rather than directly affecting emotional outcomes. As McCaffery et al. [14] emphasize, health literacy has long-term implications for decision quality and self-management, but its effects on current life satisfaction are less immediate. In a cross-sectional design such as the present one, these delayed processes cannot be fully captured. Longitudinal studies with repeated assessments are needed to further clarify the mediating role of health literacy in shaping subjective health perceptions and life satisfaction.

Overall, this study shows that life satisfaction in cancer patients is shaped predominantly by subjective health status and nutrition-related factors, while health literacy and CAM exert more indirect or long-term influences. The findings underline the need for supportive care approaches that address both the physical and perceptual dimensions of patient’s health perceptions and life satisfaction, especially by alleviating eating-related symptoms and preventing severe weight loss. Such interventions should be tailored not only to maintain physical health but also to strengthen patients’ sense of control and self-efficacy. Future research should employ longitudinal and multi-center designs to disentangle temporal dynamics and mediating pathways between health literacy, nutritional factors, and subjective health perceptions. By doing so, it may become possible to develop comprehensive intervention strategies that promote psychosocial outcomes and life satisfaction in oncology care.

## Limitations

The present findings must be interpreted in light of several methodological limitations. As a cross-sectional study, the design does not permit causal inferences. It therefore remains unclear whether a poor subjective health status reduces life satisfaction, or vice versa. Temporal developments during the course of illness, such as therapy-related improvements or long-term adaptation processes, could also not be captured. Furthermore, the data collection was based entirely on patient self-reports, entailing a risk of bias due to subjective perception, recall inaccuracies, or the tendency to present oneself in a favorable light. In addition, no medical records or objective parameters were used to validate the information provided. Moreover, we could not account for socioeconomic position (e.g., employment status/income) or tumour stage, as these variables were not part of the questionnaire. These unmeasured factors may contribute to residual confounding and restrict the interpretability of the identified associations across clinically distinct patient groups. Along the same lines, although time since diagnosis was included in the models, further stratifications by survivorship phases were beyond the scope of this manuscript. Future longitudinal studies should examine whether determinants of life satisfaction differ between early and longer-term phases following diagnosis. Another aspect concerns the potential for selection bias: as participation was voluntary, particularly engaged or more medically stable cancer patients may be overrepresented. In particular, the indirect and long-term effects of health literacy on psychosocial outcomes can only be insufficiently captured in a cross-sectional design. Future longitudinal studies are required to better disentangle these mediated pathways, for example between health literacy, subjective health perceptions, and life satisfaction. Taken together, these limitations restrict the generalizability and interpretability of individual findings. Nevertheless, the study provides important insights into the interplay of subjective health perceptions, nutritional aspects, and psychosocial outcomes in oncology patients. By systematically combining health literacy, nutrition-related factors, and patient-reported outcomes, it contributes to a more comprehensive understanding of determinants of life satisfaction in cancer. Despite the methodological constraints, the findings may thus serve as a valuable basis for hypothesis generation and for informing future longitudinal and multi-center research.

## Conclusion

This study identified subjectively perceived health status as the most important determinant of life satisfaction in cancer patients. Beyond being the strongest predictor in regression analysis, subjective health also reflected key psychosocial dimensions, including emotional stability, self-efficacy, and social participation. Physical indicators such as appetite, nutrition-related symptoms, and moderate weight changes were also associated with life satisfaction, underscoring the complex interplay between physical condition, subjective perception, and psychosocial adaptation.

These findings emphasize the need for a holistic approach to oncological care that integrates both functional and subjective dimensions. Nutritional symptoms and unintentional weight loss should be systematically assessed and managed, as they can impair physical functioning and negatively shape patients’ perception of health and overall life satisfaction. By contrast, moderate weight changes, when experienced as part of intentional lifestyle modifications, may contribute to self-regulation and coping. Recognizing this differentiated perspective may support the development of more tailored supportive care strategies.

Neither health literacy nor the use of CAM showed a direct association with life satisfaction, although health literacy appeared to exert an indirect influence through subjective health evaluation. This suggests that their effects unfold over longer trajectories via improved coping skills, health behaviors, and system navigation. Future research should therefore adopt longitudinal designs that explicitly integrate both subjective and objective health dimensions. Conceptualizing life satisfaction not as a static endpoint but as a dynamic trajectory may enable the design of patient-centered interventions that better capture the lived realities of individuals with cancer.

## Supplementary Information

Below is the link to the electronic supplementary material.

Supplementary Material 1.

## Data Availability

The datasets generated and/or analyzed during the current study are available from the corresponding author on reasonable request.

## Abbreviations

CAM: Complementary and alternative medicine
QoL: Quality of life
