# Supplementary figures and images for: Health literacy, nutrition, complementary medicine and their associations with life satisfaction in cancer patients: a cross-sectional study
Source: BMC Cancer. 2026 Mar 13;26:453. doi: 10.1186/s12885-026-15848-z (PMC13063638; doi:10.1186/s12885-026-15848-z)

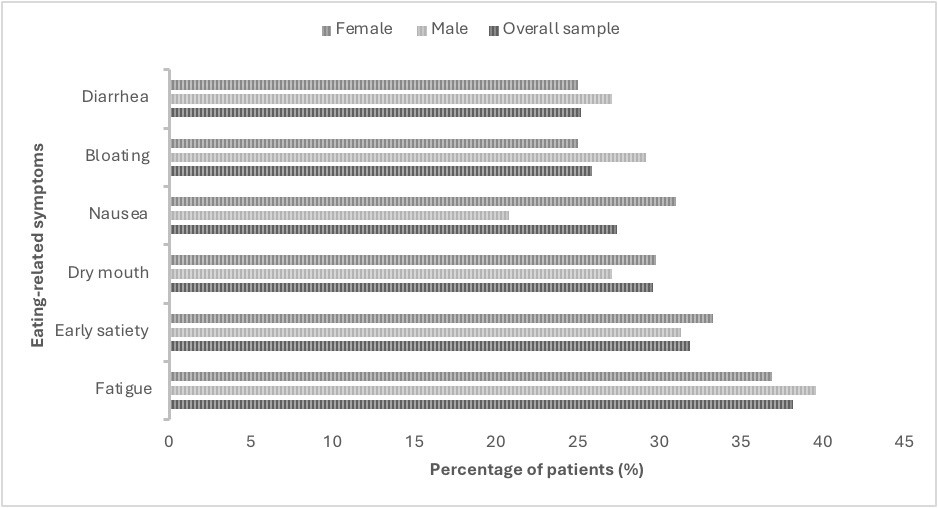

Supplement: Supplementary file 1 — Supplementary Material 1. [file 12885_2026_15848_MOESM1_ESM.jpg]
